# Socio-Demographic Factors, Behaviors, Motivations, and Attitudes in Food Waste Management of Romanian Households

**DOI:** 10.3390/nu16162738

**Published:** 2024-08-16

**Authors:** Elena Moroșan, Adriana Dărăban, Violeta Popovici, Andreea Rusu, Elena Iuliana Ilie, Monica Licu, Oana Karampelas, Dumitru Lupuliasa, Emma Adriana Ozon, Vanessa Maria Maravela, Ioana Andreea Popescu

**Affiliations:** 1 Department of Clinical Laboratory and Food Safety, Faculty of Pharmacy, “Carol Davila” University of Medicine and Pharmacy, 020956 Bucharest, Romania; elena.morosan@umfcd.ro; 2 Faculty of Pharmacy, “Vasile Goldiș” Western University of Arad, 310045 Arad, Romania; daraban.adriana@uvvg.ro; 3 Center for Mountain Economics, “Costin C. Kiriţescu” National Institute of Economic Research (INCE-CEMONT), Romanian Academy, 725700 Vatra-Dornei, Romania; 4 Faculty of Pharmacy, University of Medicine and Pharmacy “Carol Davila”, 020956 Bucharest, Romania; elena.ionita@drd.umfcd.ro (E.I.I.); vanessa-maria.maravela2022@stud.umfcd.ro (V.M.M.); 5 Department of Medical Psychology, Faculty of Medicine, “Carol Davila” University of Medicine and Pharmacy, 050474 Bucharest, Romania; monica.licu@umfcd.ro; 6 Department of Pharmaceutical Technology and Biopharmacy, Faculty of Pharmacy, “Carol Davila” University of Medicine and Pharmacy, 020956 Bucharest, Romania; oana.karampelas@umfcd.ro (O.K.); dumitru.lupuliasa@umfcd.ro (D.L.); emma.budura@umfcd.ro (E.A.O.); andreea-ioana.popescu@umfcd.ro (I.A.P.)

**Keywords:** household food waste, food purchase behavior, home food cooking, food waste frequency, homemade food waste, food waste interest, food waste awareness

## Abstract

(1) Background: Food waste (FW) in Romania is 70 Kg/capita/year, while 70% of food waste comes from public catering, retail services, and households (over 50%–47 million tons). The present study investigates the socio-demographic factors, behaviors, motivations, and attitudes related to food waste management in Romanian households. (2) Methods: A cross-sectional observational study was conducted using an online questionnaire via the Google Forms platform from 15 April 2023 to 15 May 2023. The questionnaire was designed to assess various aspects, such as some socio-demographic information (age, sex, occupation, area of residence, study level, household members number, children <18 years of age); the personal involvement and frequency of food purchases and homemade food cooking; the main sources that generate food waste; the motivation and frequency with which food waste occurs; the level of awareness regarding the impact of food waste; the respondents’ intentions regarding sustainable behaviors and practices for food management; the level of information and familiarity of the respondents with the notions of validity and how these may influence their food consumption decisions. (3) Results: The results show that FW incidence is occasionally (42%), very rarely (43.33%), frequently (15%), and no food waste was reported by 2.66% of respondents. The 35–44 age category records the highest FW frequency, followed by 18–24. The most wasted are homemade food (29.67%), bread and bakery products (27.00%), and fruits and vegetables (14.33%). High involvement in purchasing and buying food following a previously established list reduces FW frequency. The same is valid for high daily involvement in food and homemade cooking. High interest in the FW problem and its perception as a waste of money leads to diminishing it, while guilty feelings increase the FW level (37.50% to 73.33%). (4) Conclusions: The present study shows that household food waste management is a multifactorial process that involves numerous socio-demographic, behavioral, and emotional aspects. Extensive data analysis supports our results, revealing deep self-reported information details and confirming its complex approach.

## 1. Introduction

Nutrition consists of food intake directly related to the body’s needs. Life is maintained with food; it contains nutrients essential for growth, functionality [1], protection [2,3], restoration of body issues [4,5], and regulation of metabolic processes in health and disease [6,7,8]. Moreover, food is deeply integrated into people’s social life. Homemade foods, for example, could be expressions of personal feelings that highlight interhuman relationships. Preparing and eating specific food are traditional aspects of various communities during the most significant times in life [9,10].

From farm to fork, a complex system involves linked processes of production, aggregation, processing, packaging, distribution, and disposal of final food products to potential consumers. However, while 9.2% of the global population (about 700 million people) live in extreme poverty and cannot afford daily food to survive, a third part of the food produced in the world is lost or wasted [11]. FAO estimates that over 30–40% of total food production can be lost before market distribution due to inappropriate post-harvest storage, processing, or transportation (over 40–50% of root crops, fruits, and vegetables, 30% of cereals and fish, and 20% of oilseeds). Other causes consist of food overproduction, which exceeds demands due to the availability of crop subsidies, and the safe food removal from the market or supermarket shelves due to urgent regulations [11]. Finally, consumers who buy more food than their families can eat and their household habits could be other important factors that lead to food waste (FW). Therefore, the FAO proposed Save Food [12], a global initiative involving numerous public and private sector partners in reducing loss and waste [11].

The United Nations General Assembly (UNGA) marked September 29 as the International Day of Awareness of Food Loss and Waste (IDAFLW). This day significantly contributes to highlighting the emerging FW problem [12]. Eurostat 2023 reported that around 10% of food for EU consumers (at retail, food services, and households) could be wasted. Over 37 million people cannot afford a quality meal every second day. UNEP Food Waste Index 2024 shows that around 1.05 billion tons of food waste were generated in 2022: 12% from retail, 28% from food services, and 60% from households. Considering FW per capita/year, Greece is in the highest position with 142 Kg, and Russia in the lowest, with 33 kg [13], according to the United Nations Environment Program report from 2021.

Reducing FW is a substantial support against climate change because it has an enormous environmental impact, generating 16% of the total Greenhouse Gas emissions from the EU food system. Moreover, saving nutritive food for those in need, helping to eradicate hunger and malnutrition, and preserving money for households, companies, and farms could be other essential benefits of FW diminution [12].

Celebrated annually in Romania on October 16, the National Day of Food and Combating Food Waste is an opportunity to remember that lack of food, hunger, and malnutrition can affect any country in the world and that global actions are needed to reduce the amount of wasted food. In Romania, Law No. 217/2016 on reducing food waste regulates the activity of economic operators in the agri-food sector. According to this law, food waste is when food leaves the human consumption circle due to degradation and is destroyed. Following the current legislation, consumers have access to reduced prices for food before they expire, and the procedure for food donations to non-governmental organizations has been simplified [14]. Eurostat reports that Romanian households allocated 26.4% of total consumption expenditure to food and non-alcoholic beverages in 2020, while in the EU, the average is 14.8%. More than 4 million Romanians have difficulties in daily food purchases, while every year, a third of the food purchased is thrown away; food waste for our country was 70 Kg/per capita/year (as for the Czech Republic and Slovakia), estimated to be 1,353,077 tons/year, according to the report of the United Nations Environment Program [13] from 2021. On average, 70% of food waste comes from public catering, retail services, and households (over 50%–47 million tons) [13]. Thus, household food waste is one of the major obstacles to meeting global emission targets, and its level significantly varies within a year. For example, holiday marketing campaigns promote images of lavish meals and stocking up on various delicacies [15] compared with the rest of the year. Thus, people buy more than they need or prepare exaggerated portions [16,17]. These practices often result in a wide variety of foods on the table, which may not be fully consumed. Lack of planning or knowledge of how to store and recycle food can lead to it being thrown away instead of being used effectively.

Therefore, it is still hard to control the FW from households [18,19,20]. In this context, the present study aims to investigate the food waste in Romanian households. It also evaluates the attitudes, respondents’ behaviors, knowledge, and perceptions about food waste by obtaining direct information from the participants.

## 2. Materials and Methods

A cross-sectional observational study was conducted using an online questionnaire in the Romanian language via the Google Forms platform from 15 April 2023 to 15 May 2023. Its multiple-choice questions (with single or multiple answers) were designed to assess various aspects: (1) some socio-demographic information (age, sex, occupation, area of residence, studies, household members number, children <18 years of age); (2) the personal involvement and frequency of food purchase (FP) and homemade food (HMF) cooking; (3) the most significant sources of household food waste; (4) the causes and frequency of food waste; (5) the level of awareness regarding the FW impact; (6) the respondents’ intentions regarding sustainable behaviors and practices for food management; (7) the level of information and familiarity of the respondents with terms regarding food safety and how these may influence their food purchase and consumption decisions. The survey involved voluntary participants over 18 years old residing in Romania. Twenty-seven questions were generated in electronic format, and the research team members distributed the URL link to the survey via email or SMS to colleagues and relatives and via social networks to personal contacts. Participants were informed about the purpose of the survey, the research team involved, and the time required to complete the questionnaire; moreover, they were assured that any email address was collected and that the General Data Protection Regulation (GDPR) guarantees the confidentiality of sensitive personal information. Then, they completed and signed the participation agreement and the individual consent form to enable the publication of research results. Three hundred respondents correctly completed the questionnaire in the previously mentioned period. All data were recorded anonymously, and the database was assessed using a Microsoft 365 Excel v. 2024 workbook.

### Data Analysis

Statistical analysis was performed using XLSTAT Life Sciences 2024.1.0. 1418 by Lumivero (Denver, CO, USA) [6,21,22]. The questionnaire was investigated using Reliability Analysis from XLSTAT. Cronbach’s alpha index, Spearman–Brown coefficient, and Guttman L4 coefficient were calculated, and their values were over 0.9. The results were included in Table S1 from Supplementary Materials.

All variables are displayed using absolute frequencies (N) and relative frequencies (%). The needs, preferences, and various diet types depend on the age of individuals. Several complex responses were simplified, briefly maintaining the essential information to facilitate statistical analysis.

Extensive data analysis used different tools of XLSTAT Life Sciences: descriptive analysis, ANOVA single factor, the Kruskal–Wallis samples comparison, correlations between variable parameters through Principal Component Analysis, and heat maps. Statistical significance was established at *p* < 0.05.

## 3. Results

### 3.1. Socio-Demographic Data

Table 1 shows that, of the 300 respondents, 81.66% are female, 18.33% are male, 88.66% live in urban areas, and 11.33% live in rural zones. Considering their age, 34.33% are between 35 and 44 years, 32.66%—18 and 24 years, 15.66%—45 and 54 years, 11%—25 and 34 years, 4%—45 and 54 years, and 2.33%—≥65 years. Regarding the educational level of participants, 57.33% have a bachelor’s degree, 27.33% are postgraduates, 12% have high school education, and 3.33% have post-high school studies. A total of 18.33% of participants live alone, while, in 31% of cases, the household has 2 members; 28.33%—3 members, 18%—4 members, and 7.33%—≥5 members; 55.33% of families have children under 18.

**Figure 1:**
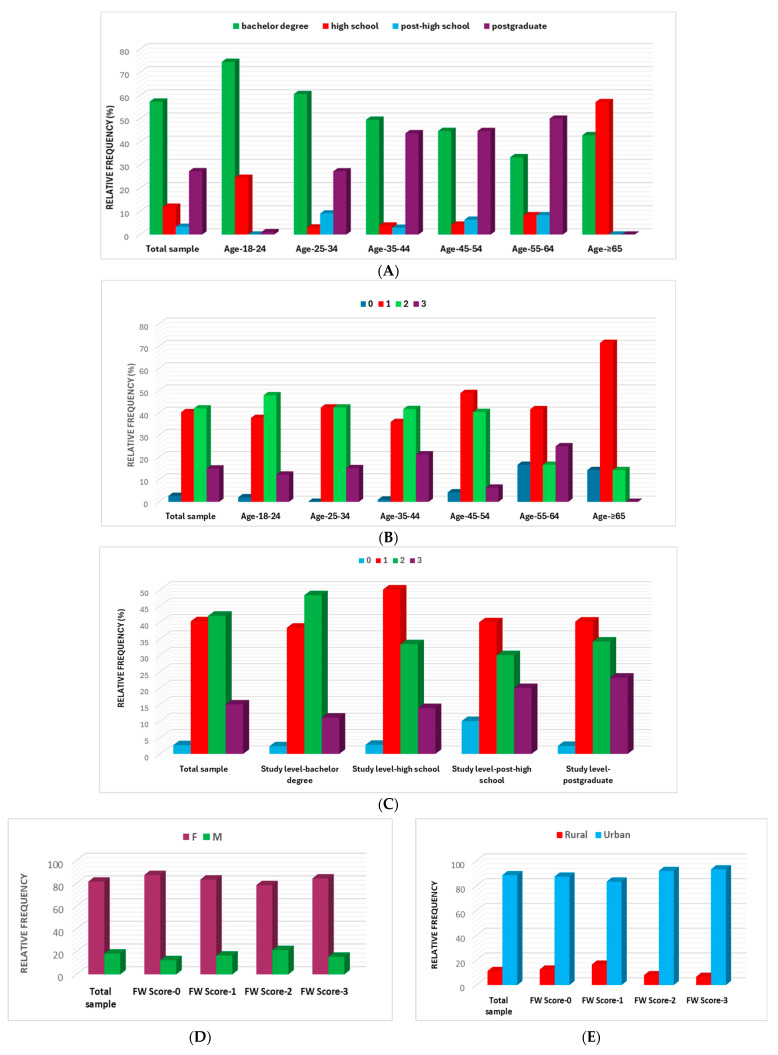

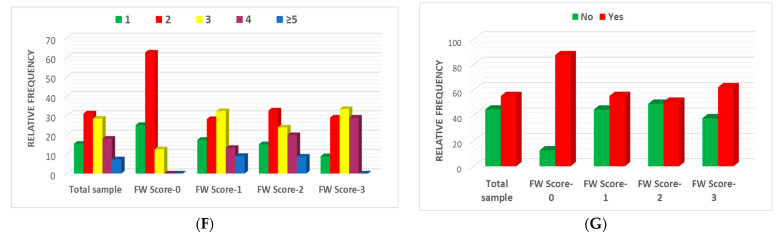
(**A**) Study levels and the age of participants. (**B**–**G**) Socio-demographic factors and FW frequency: (**B**) age, (**C**) study level, (**D**) sex (F−female, M−male); (**E**) residence zone (rural and urban); (**F**) family members number; and (**G**) children < 18 years.

Data registered in Table 1 evidence that FW incidence is occasionally (42%), very rarely (43.33%), and frequently (15%); 2.66% of respondents stated that they never waste food. Therefore, considering FW level expressed by the frequency of throwing foods, four distinct groups were identified and marked with the following scores: FW-0 (never, no FW), FW-1 (very rarely, mild FW), FW-2 (occasionally, moderate FW), and FW-3 (frequently, high FW). All socio-demographic data from Table 1 were described, reported for FW frequency, and compared with the total cohort (Figure 1).

Over 80% of the respondents have the highest study levels (bachelor’s degree and postgraduate); they are similarly distributed in the 35–54 age groups (49.51% and 44.68% vs. 43.69% and 44.68%). A total of 50% of the 55–64 are postgraduates (Figure 1A). The lowest FW levels (FW-0 + FW-1) were recorded in the > 65-year group (85.71%), followed by 55–64 and 45–54 (58.33% and 53.19%). The highest FW levels (FW-2 + FW-3) were recorded in the 35–44 group (63%) followed by the 18–24 and 25–34 groups (60.20% and 57.50%). The highest study levels are associated with substantial FW: bachelor degree and postgraduate (59.3% and 57.32%) vs. post-high school and high school (50% and 47.22%) (Figure 1B,C). Moreover, the total cohort’s FW level is high (FW-2 + FW-3 = 57%). Figure 1D associates high FW levels with male presence (61.81%) vs. female (55.91%). In the rural zone, the FW level is significantly lower than the urban one (38.23% vs. 59.39%), as indicated in Figure 1E. Figure 1F associates FW-0 with 1–3 household members. Generally, the FW level of all households is ≥50%, with a maximal value of 70.37% for 4 household members. Figure 1G shows that food waste is lower in a household with children <18 years (38.55% vs. 58.95%)

**Figure 2:**
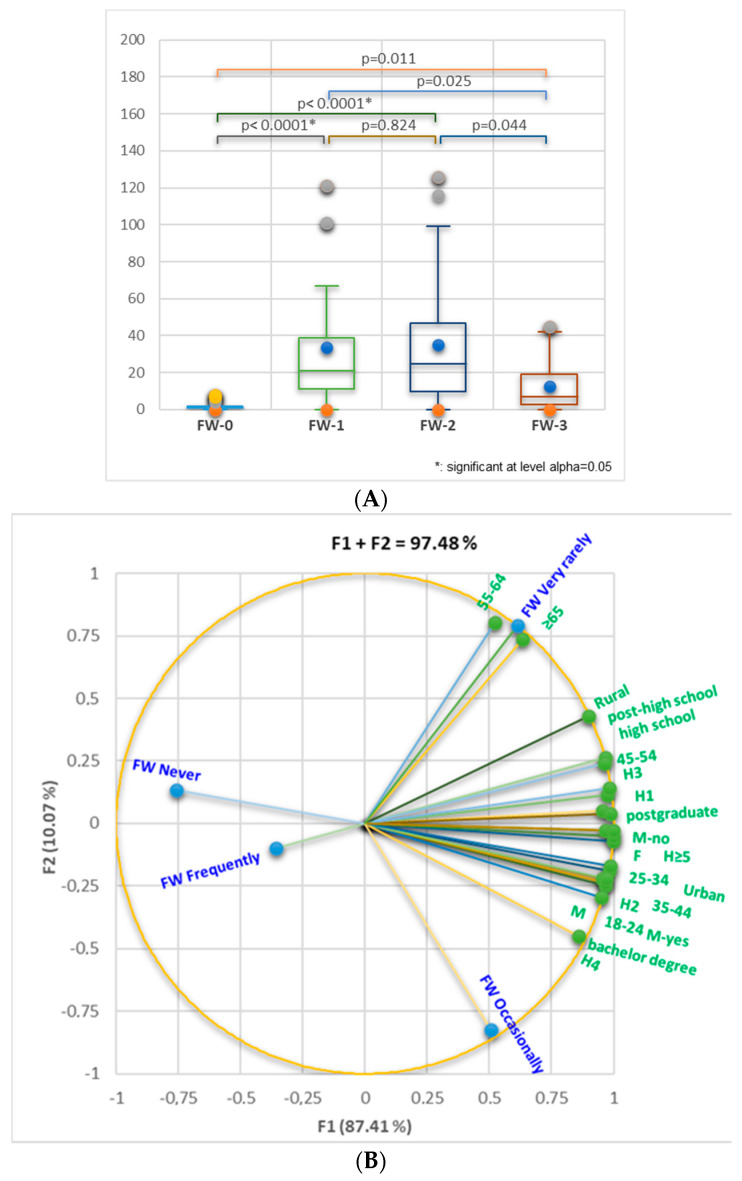
(**A**) Kruskal–Wallis analysis for FW-0, FW-1, FW-2, and FW-3 comparison considers general data of respondents. Bonferroni-corrected significance level = 0.0083. (**B**) Correlation between age groups (18–24, 25–34, 35–44, 45–54, 55–64, ≥65), gender (male (M)/female (F)), education level (high school, residence, number of household members (H1—H ≥ 5), the presence of children (minors <18 years, M-yes/M-no), residence (rural/urban), and the FW-level (FW-0—FW-3).

Statistical differences are recorded between FW-0 vs. FW-1 and FW-0 vs. FW-2 (*p* < 0.0001), as Figure 2A shows. The correlation between the cohort’s general data and FW level is displayed in Figure 2B. The total data variance is 97.20%. The FW levels and all variables belong to the F1 axis. Only four ones are associated with F2. Figure 2B indicates that FW-1 (very rarely) significantly correlates with the age group ≥65 (*r* = 0.977, *p* < 0.05); FW-1 also shows a strong correlation with the 55–64 age group and rural zone, and a moderate one with high and post-high school education, H3, and 45–54 age group (*r* = 0.943, *r* = 0.893, *r* = 0.797, *r* = 0.775, *r* = 0.707, *r* = 0.694, *p* > 0.05). FW-2 (occasionally) moderately correlates with male gender, H4 and H2, 18–24 and 35–44 age groups, M-yes, bachelor’s degree, and urban zone (*r* = 0.744, *r* = 0.741, *r* = 0.714, *r* = 0.711, *r* = 0.695, *r* = 0.650, *r* = 0.648, *r* = 0.614, *p* > 0.05).

### 3.2. Food Purchase and Home Cooking Behavior

**Figure 3:**
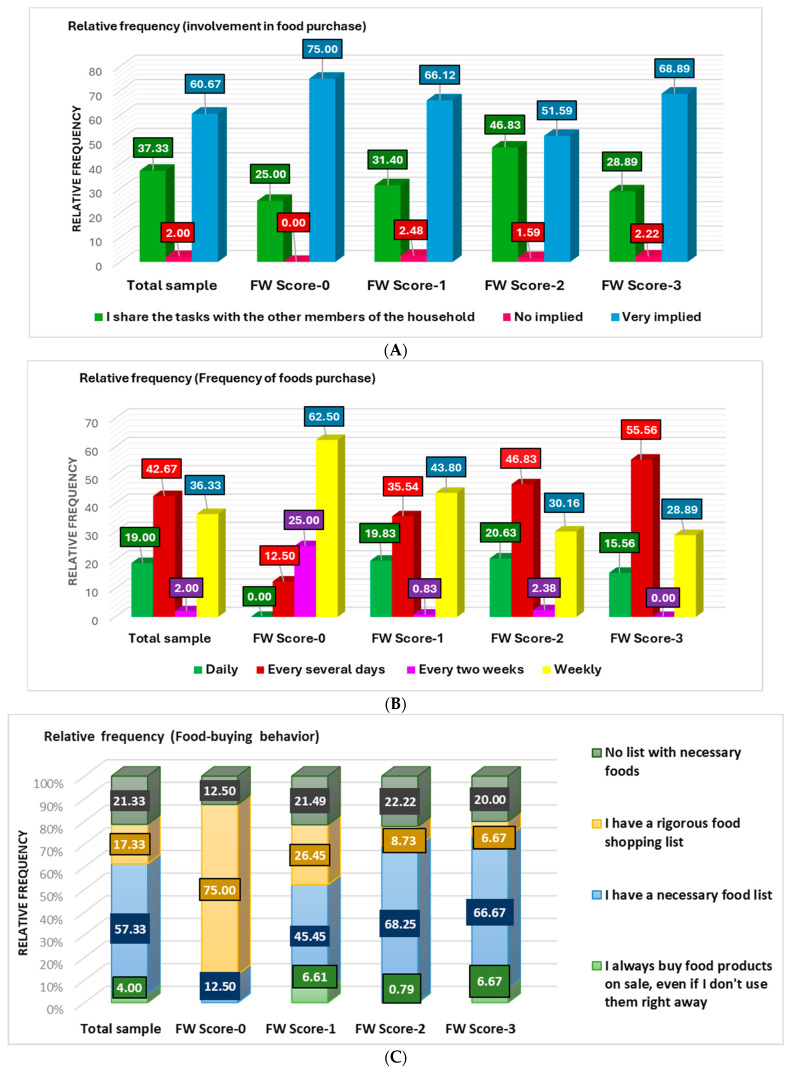

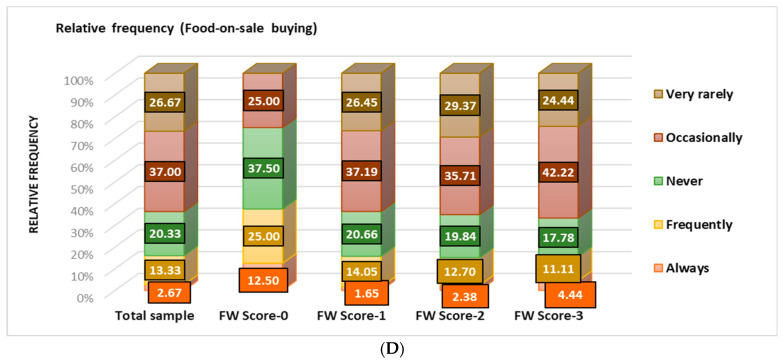
Food purchase (FP) behavior correspondence to FW score: (**A**) involvement in food purchase; (**B**) frequency of food purchase; (**C**) food buying behavior; (**D**) food-on-sale buying.

All data obtained through descriptive analysis and grouped following FW-score are registered in Table S2 and illustrated in Figure 3.

From the cohort, 60.67% declared they are very involved in food purchases, 37.33% share this activity with other household members, and 2% are uninvolved (Figure 3A).

Only 19% of participants buy food daily, and 36.33% opt for it once a week (Figure 3A). The highest percentage (42.67%) buy food for several days. Only 2% reported the most reduced frequency—every 2 weeks (Figure 3A), most (25%) belonging to the FW-0 group. Over 50% of participants (57.33%) have a list of necessary food (Figure 3B). Only 17.33% make food acquisition according to a rigorous list; 75% of the FW-0 group proceed in this mode. The list is unnecessary for 21.33% of respondents; they belong to FW-1, FW-2, and FW-3 groups in similar percentages (20–22.22%). Only 4% of participants always buy food on sale (OSF, Figure 3C); a relatively similar value is indicated by Figure 3D (2.67%). A total of 88% of participants said they understood the concept “preferably consumed before” (Table S2). However, 13.33% frequently buy OSF, 36% occasionally, 26.6% rarely prefer it, and only 20.33% never chose this food category. A total of 53.67% are very implied in-home cooking, and 52.33% prepare homemade food 2–3 times a week (Figure 1E,F); Table S2 indicates the most significant users: 75% of the FW-0 group are very implied in HC, and 68.89% of FW-3 prefer to cook 2–3 times a week.

**Figure 4:**
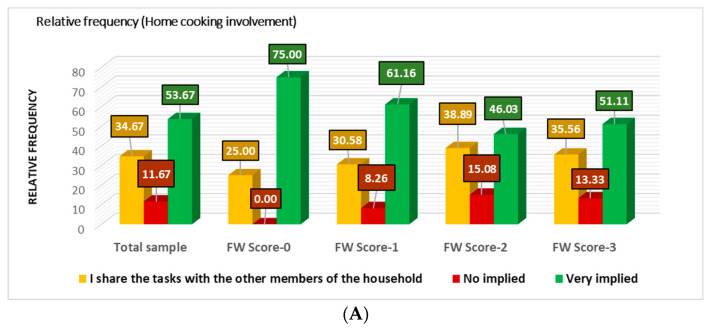

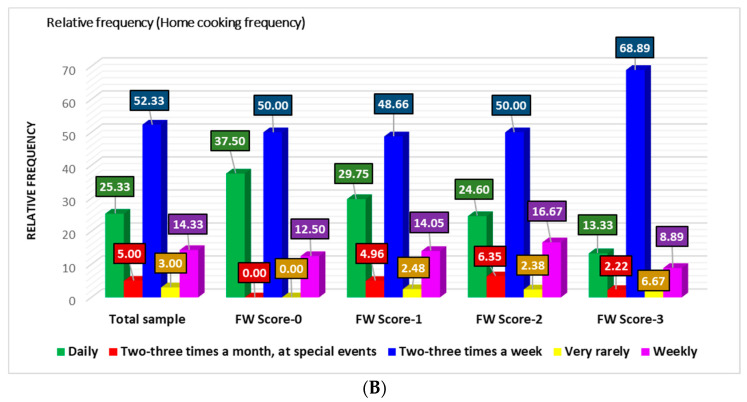
Home cooking (HC) behavior in concordance with FW frequency: (**A**) home cooking involvement; (**B**) home cooking frequency.

In the present study, 53.67% of the cohort is involved in home cooking, 34.67% shares it with other family members, and 11.67% are not implied. Most respondents of FW-0 and FW-1 groups are implied in FC (75% and 61.16%), and only 8.26% of FW-1 are not implied (Figure 4A).

**Figure 5:**
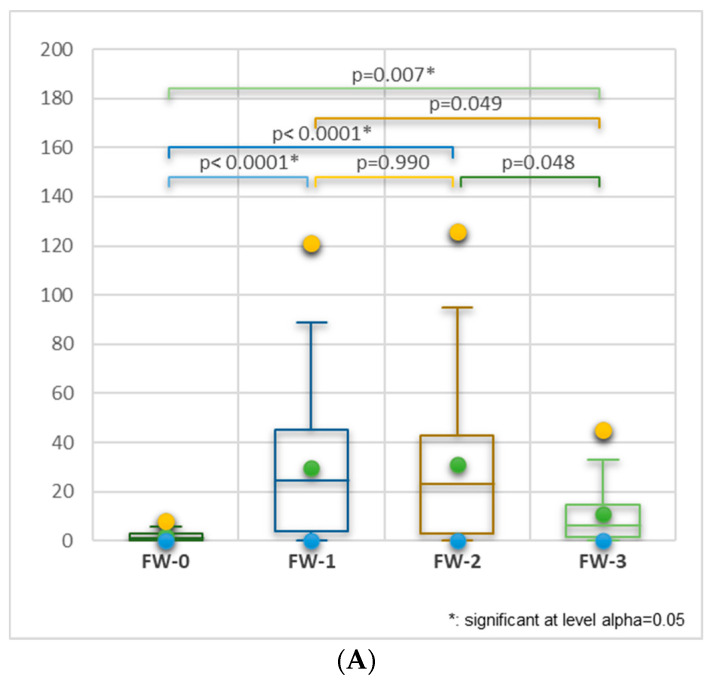

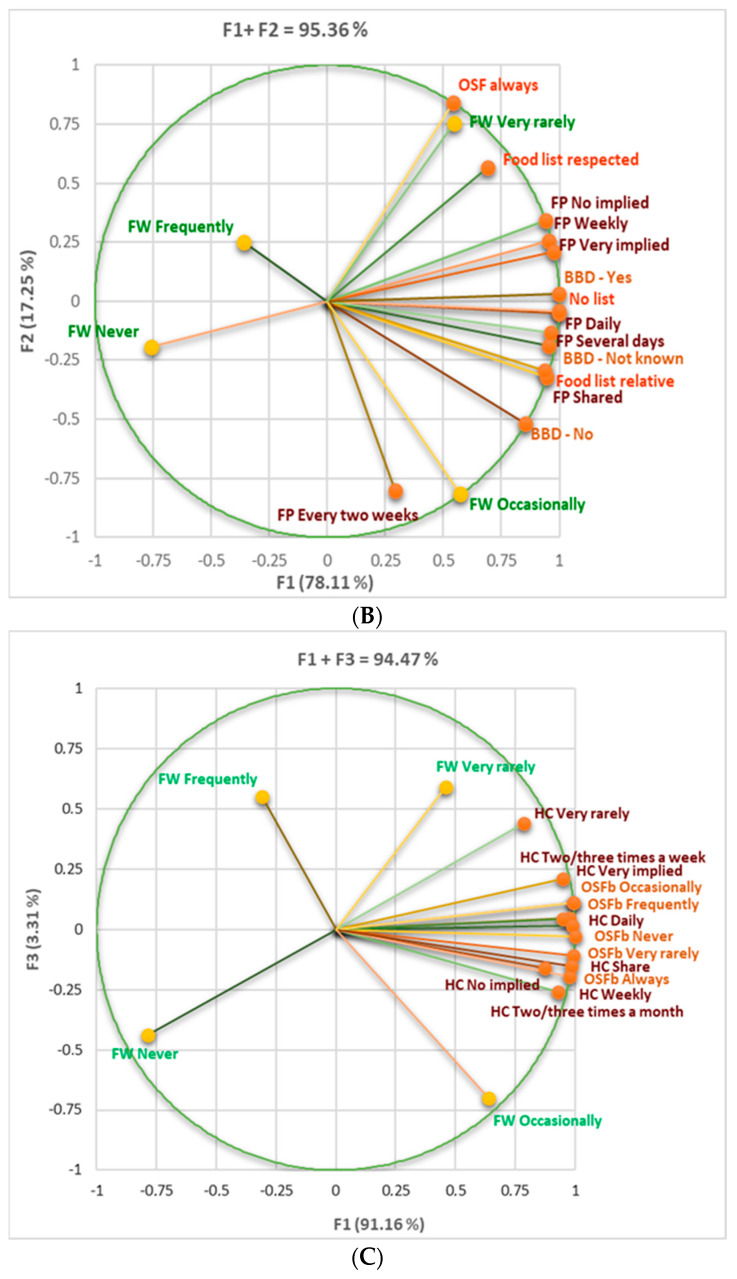
(**A**) Kruskal–Wallis’s analysis for FW-0, FW-1, FW-2, and FW-3 comparison considers food buying and homemade food cooking behavior data. Bonferroni-corrected significance level = 0.0083. (**B**) Correlation between FW-level (FW-0–FW-3) and food purchase (FP) aspects: involvement in FP, food-on-sale buying preference, FP frequency, food buying behavior, and BBD knowledge status; (**C**) correlation between food cooking aspects: frequency, involvement, OSFb preference; FP = food purchase, FC = food cooking, BBD = best before date, OSF = food-on-sale (with a low BBD), OSFb = food-on-sale buying; HC = home cooking, FW = food waste.

The analysis of data registered in Table S2 is illustrated in Figure 5.

Figure 5A shows the following statistically significant differences: FW-0 vs. FW-1, FW-0 vs. FW-2 (*p* < 0.0001), and FW-0 vs. FW-3 (*p* = 0.007). Figure 5B and the correlation matrix (Supplementary Materials) have shown a significant correlation (*r* = 0.968, *p* < 0.05) between FP with a rigorously respected list and FW-1. BBD-No and FP sharing is highly correlated with the FW-2 group (*r* = 0.905, *r* = 0.808, *p* > 0.05), and OFSb-always strongly correlates with FW-1 (*r* = 0.937, *p*> 5). FP weekly and FP-no implied moderately correlate with FW-1 (*r* = 0.774, *r* = 0.775, *p* > 0.05), while FP frequency (every two and several days) and food list relative are moderately correlated with FW-2 (*r* = 0.723—0.792, *p* > 0.05). Variables linked to FP frequency, FP involvement, and needed food lists are negatively correlated with FW-0 (*r* = −[0.744—0.830], *p* > 0.05), while FP every 2 days shows a moderate negative correlation with FW-3 (*r* = −0.775, *p* > 0.05).

According to Figure 5C, FW-0 reports a substantial negative correlation (*r* = −0.999, *p* < 0.05) with HC very rarely and a good one with HC two or three times a week, OSFb always and occasionally (*r* = −[0.816–0.855], *p* > 0.05). FW-2 is strongly correlated with OSFb always, and FC is not implied (*r* = 0.816, *p* > 0.05). As an overview, food buying following a rigorously respected food list led to very rarely waste food (FW-1); the participants from this category prefer to buy food-on-sale (with a low BBD). The respondents with no BBD knowledge add that those who share the FP action with other family members belong to the FW-2 group.

### 3.3. Homemade Food Leftovers Management and FW Status Investigation

All data are classified according to FW score and registered in Table S3. A total of 82.33% of the respondents prefer to save HFL for the next day; they represent 79.34% of FW-1 and FW-2 groups, 75.56% of FW-3, and 75% of FW-0. Only 3.33% throw away HFL, corresponding to 5.56% FW-2 and 6.67% FW-3 groups. No leftovers are reported by 25% FW-0, 10.74% FW-1, and 2.38% FW-2, summing 6% of the total cohort. Only 1.67% prefer to freeze HFL; all belong to the FW-1 group (4.13%). Only 2.67% of respondents categorically stated that they never waste food; all others (87.33%) waste food with different frequencies (rarely, occasionally, and frequently).

**Figure 6:**
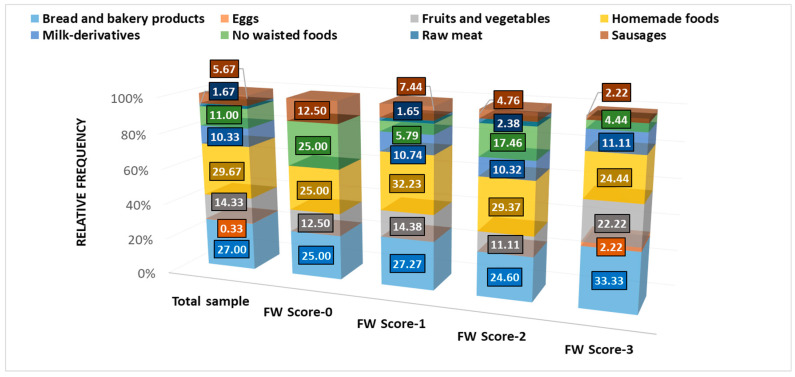
The most frequently wasted food—expressed as a relative frequency (%) in each FW group compared to the total cohort.

Therefore, our survey aims to investigate the most wasted foods (Figure 6) and the principal factors that lead to this process.

Figure 6 indicates that 25% of FW-0 group participants stated “no wasted foods”, followed in decreasing order by the FW-2 group (17.46%), FW-1 (5.79%), and FW-3 (4.44%); all represent 11% of the total cohort.

The highest percentages of wasted food are represented by homemade food (29.67% of the total cohort, 32.23% of FW-1) and bread and bakery products (27.00% of the total cohort, 33.33% of FW-3). Then, 14.33% of the total respondents wasted fruits and vegetables, FW-3 having the highest percentage (22.22%). Only 10.33% of the total attendants wasted milk derivatives, with similar percentages in FW-1, 2, and 3 groups (10.74%, 10.32%, and 11.11%). Sausages are wasted by 5.67% of the total cohort; FW-0 has the highest percentage (12.50%). Only 1.67% of respondents waste raw meat; they belong to FW-1 (1.65%) and FW-2 (2.38%). Finally, 2.22% of the FW-3 group waste eggs, representing 0.33% of the total cohort.

**Figure 7:**
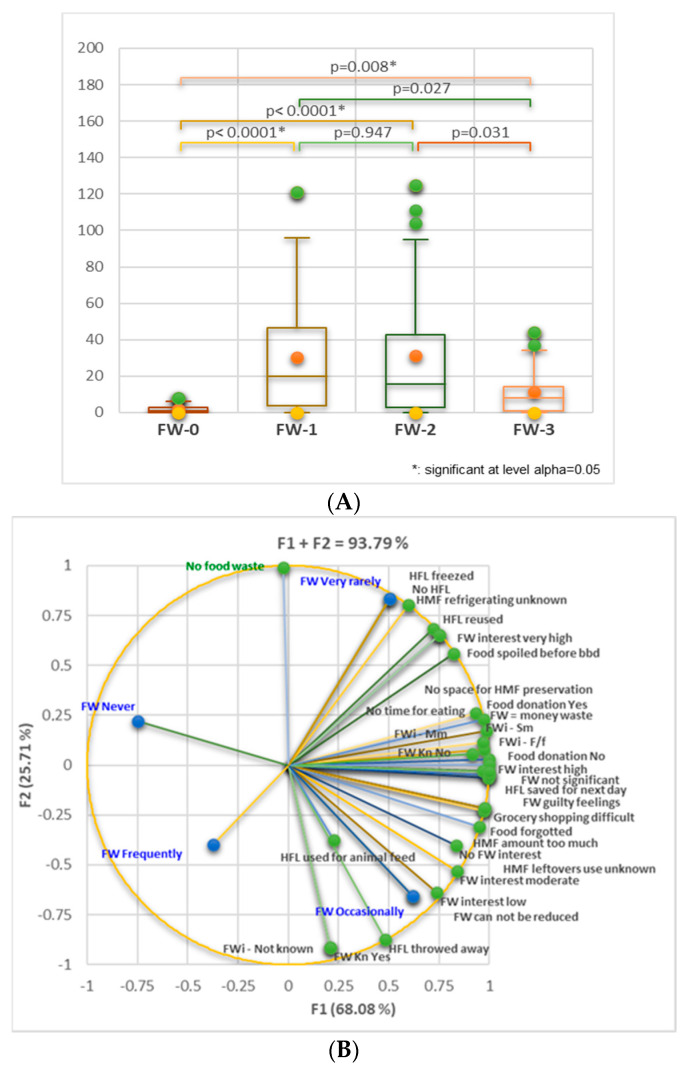
(**A**) Kruskal–Wallis’s analysis for FW-0, FW-1, FW-2, and FW-3 comparison considers all data related to FW, registered in Table 1. Bonferroni-corrected significance level = 0.0083. (**B**) Correlation between FW-level (FW-0—FW-3) and the aspects involved in FW. FW = food waste; kn = knowledge; FWi = food waste information; F/f = family and friends; Mm = mass media; Sm = social media; HMF = homemade food; HFL = homemade food leftovers.

Figure 7A indicates significant differences between FW-0, FW-1 (*p* < 0.0001), and FW-2 (*p* = 0.008). Figure 7B shows that FW very rarely (FW-1) is significantly correlated with HFL froze and no-HFL (*r* = 0.999, 0.977, *p* < 0.05), HFL reused (r = 0.943, *p* > 0.05), and FW interest very high (*r* = 0.906, *p* > 0.05). FW occasionally (FW-2) substantially correlates with FW low interest (*r* = 0.943, *p* < 0.05) and no FW interest (*r* = 0.943, *p* > 0.05). FW-2 moderately correlates with “No time for eating” (*r* = 0.700, *p* > 0.05), “Food forgotten”, “HMF amount too much”, “HFL use not knowing”, and “Grocery shopping is difficult” (r = 0.728–0.781, *p* > 0.05). As an overview, 82.33% prefer to save the HMF for the next day. Only 3.33% throw away food leftovers, and 2.67% declared no leftovers. According to Figure 7B, the respondents with minimal FW (very rarely) show great interest in FW and try to diminish it. They have no leftovers because they know to preserve them by freezing and reusing them. FW increases when the participants have low interest or are not interested in this process.

### 3.4. Knowledge, Feelings, Motivation, and Food Waste Frequency

**Figure 8:**
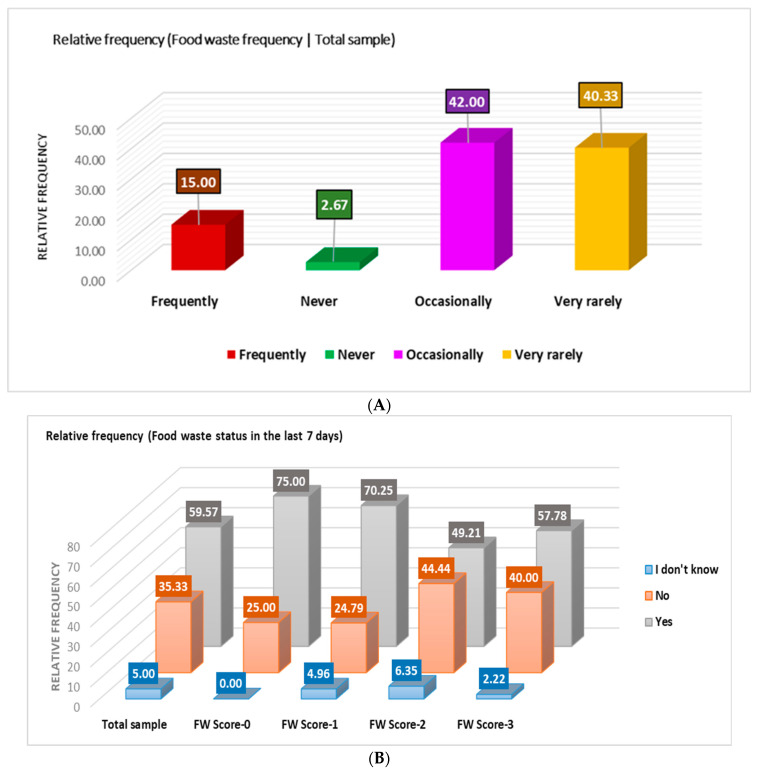
The correspondence between FW score (**A**) and FW status in the last 7 days (**B**).

A key question in the online survey investigates the FW status in the last 7 days, having only three answers available: “Yes”, “No”, and “I don’t know”. Therefore, an extensive descriptive analysis was performed on the total cohort data, separating three categories of participants according to each response to this question with all variable parameters. All data were registered in Table S4 and Figure 8.

Figure 8B shows that 59.67% of the cohort revealed food waste in the last 7 days; the highest percentage belongs to the FW-1 group (47.49%). However, of the eight respondents in the FW-0 group, six (75%) gave a positive answer. Therefore, the self-reported behavior in the previous questions differs from the real one, revealed in the later and more subtle ones [23].

**Figure 9:**
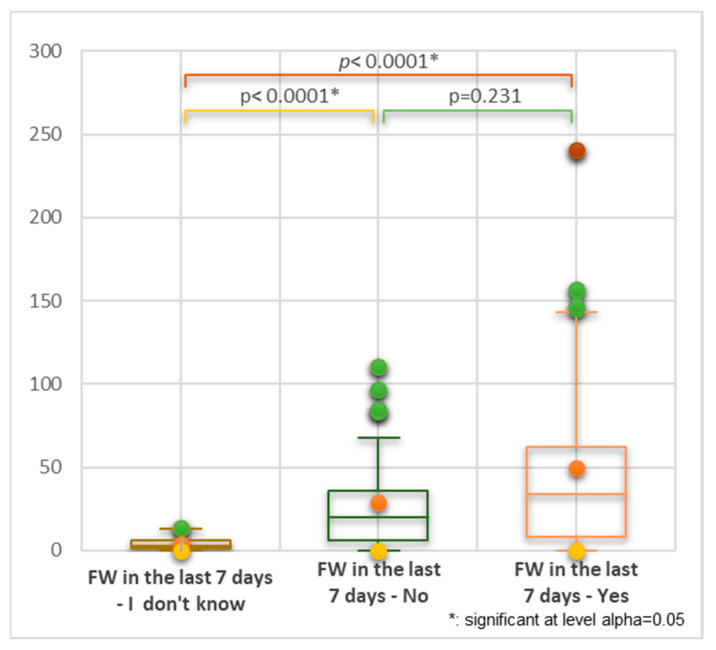
Kruskal–Wallis analysis of all three groups of FW status in the last 7 days. Bonferroni-corrected significance level = 0.0167.

All three groups recorded in Table S4 were compared using the Kruskal–Wallis analysis. No significant statistical differences between the positive-answer (Yes) and the negative-answer (No) groups were evidenced (*p* = 0.231, Figure 9).

Most respondents are female (82.12%) from the urban zone (87.71%), have a bachelor’s degree (59.78%), and are between 35 and 44 (36.31%) and 18 and 24 (33.52%) years old.

The foodbank concept is unknown to 80.33% of attendants; a similar percentage (79.89%) are not involved in food donation. Over 80% understand the concept of “preferably consumed before” and the difference between it and “to be consumed up to”.

**Figure 10:**
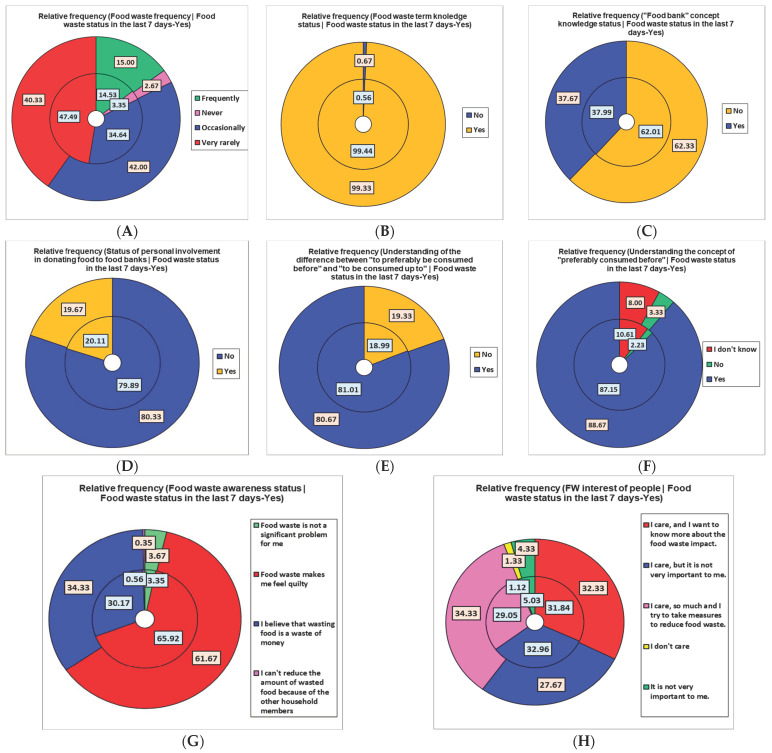

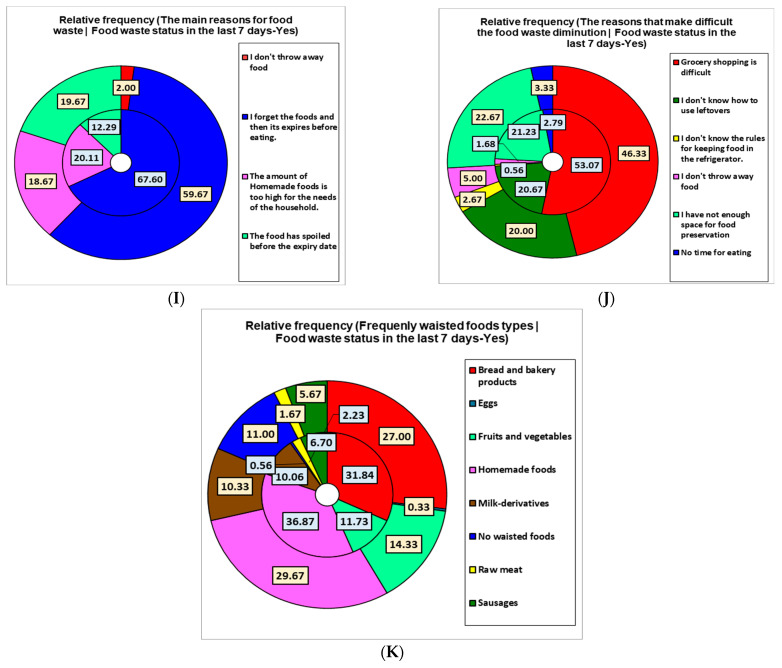
Food waste in the last 7 days’ positive status (Yes) and related variable parameters (relative frequency %) compared with the total cohort. (**A**) FW-score; (**B**) food waste term knowledge status; (**C**) foodbank concept knowledge status; (**D**) status of personal involvement in donating foods; (**E**) status of understanding the difference between “to preferably be consumed before” and “to be consumed up to”; (**F**) status of understanding the concept of “preferably consumed before”; (**G**) food waste awareness status; (**H**) status of FW personal interest; (**I**) the main reasons for food waste; (**J**) the reasons for difficulties in making food waste diminish; (**K**) frequently wasted food types.

The main reason for FW is “I forget the food, and then it expires before I eat” (67.60%); moreover, the most significant aspect against FW diminution is “Grocery shopping is difficult” (53.07%). The participants wasted food in the last 7 days, even though they care about food waste (approximately 94%) and have guilty feelings (65.92%), as Figure 10 indicates. The most wasted foods are HMF (36.87%) and bread and bakery products (31.84%).

Figure 10A shows similar relative frequencies in the targeted group vs. total cohort for FW-3 and FW-0 (14.53% and 3.35% vs. 15% and 2.67%, respectively). Higher differences were recorded in the case of FW-1 and FW-2 (47.9% and 34.64% vs. 40.33% and 42%, respectively). Positive responses regarding FW knowledge status were reported at 99.44% vs. 99.33% of the total cohort (Figure 10B). Food bank concept knowledge, the implication in donating food, understanding the difference between “BBD” and “up to”, and the concept of BBD are similar (37.99%, 20.11%, 81.01%, and 87.15% vs. 37.67%, 19.67%, 80.67% and 88.67%, respectively) (Figure 10C–F).

FW awareness status expressed as “guilty feelings” and waste money” is slowly different (65.92% and 30.17% vs. 61.97% and 34.33%, Figure 10G). FW interest status, expressed as “I care so much, and I want to take measures to reduce FW” and “I care, but it is not very important to me”, recorded a few differences (29.5% and 32.96% vs. 34.33% and 27.67%, Figure 10H). Minimal to moderate differences were remarked in the case of forgotten food (67.60% vs. 59.67%, Figure 10I), “Grocery shopping is difficult”, and “I don’t throw away food” (53.07 vs. 46.33%, respectively, and 1.68 vs. 5%, Figure 10J) and “HMF wasted” and “No wasted food” (36.87 vs. 29.67, respectively, and 0.56 vs. 11%, Figure 10K). Bread and bakery products and homemade foods are greatly wasted (31.84% and 36.87% vs. 27% and 29.7%, Figure 10K).

### 3.5. Correlations between Socio-Demographic Data, FP, HC, and FW Aspects

**Figure 11:**
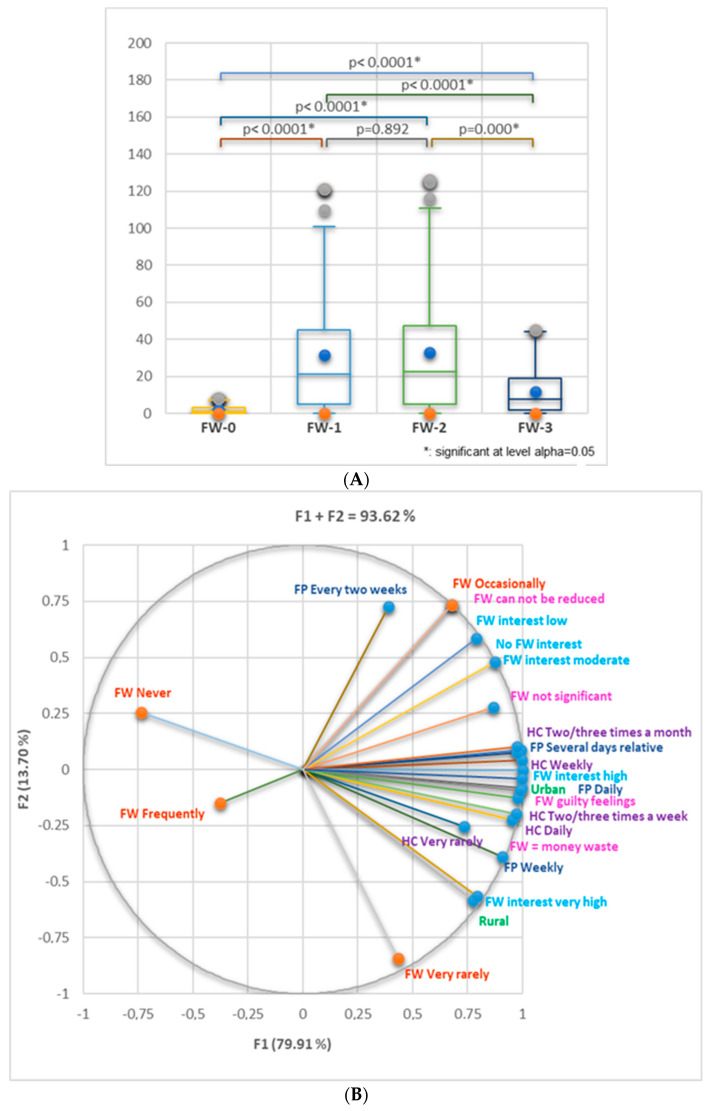

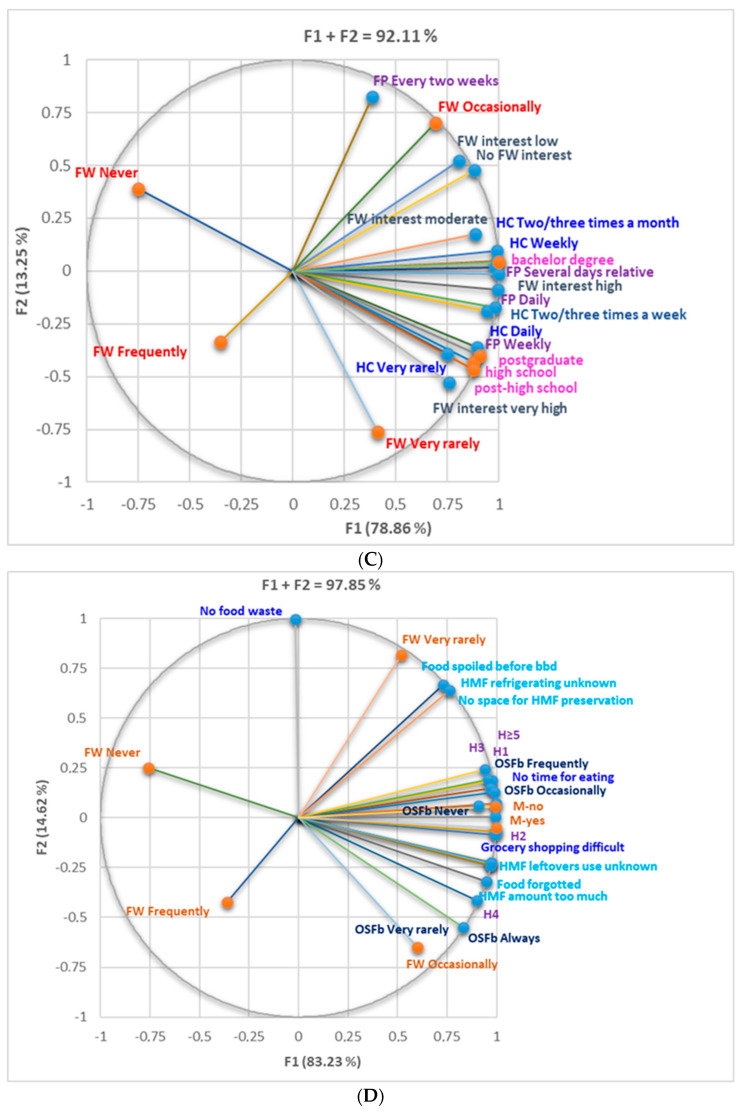

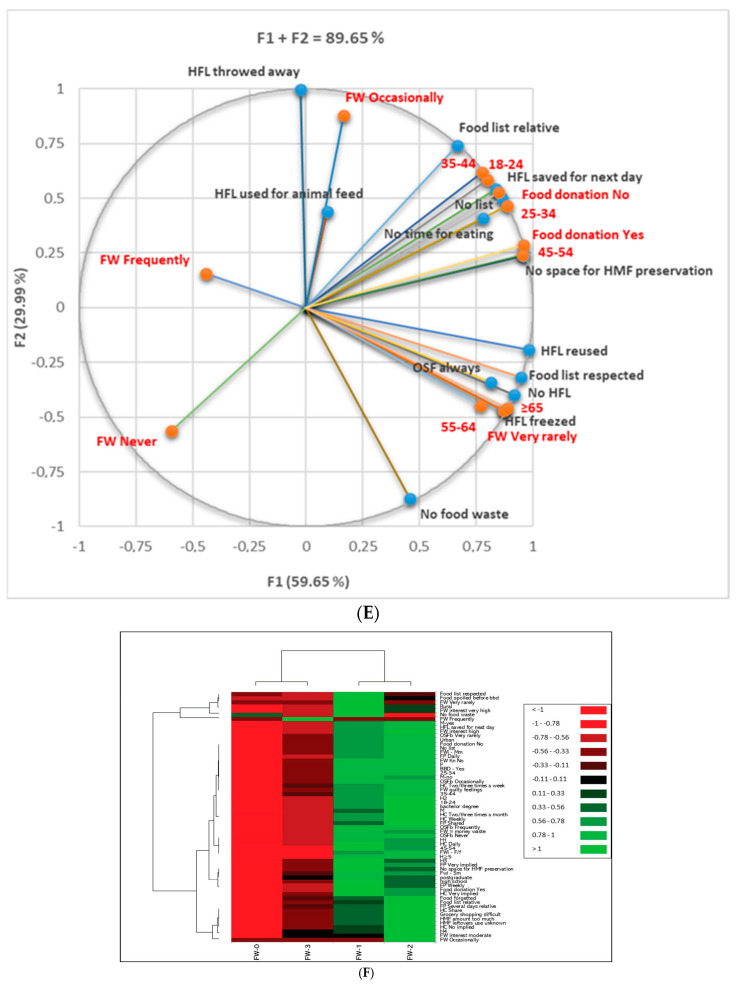
(**A**) Kruskal–Wallis’s FW-0, FW-1, FW-2, and FW-3 comparison considers all data (general and related to FP and FW) registered in Table 1, Tables S2 and S3. Bonferroni-corrected significance level = 0.0083. (**B**–**E**) Correlation between FW-level (FW-0—FW-3), general data, and the aspects involved in FP and FW. (**F**) Heat maps with significant differences between all FW-scored groups. FW = food waste; kn = knowledge; FWi = food waste information; F/f = family and friends, Mm = mass media; Sm = social media; HMF = homemade food; HFL = homemade food leftovers; H1-H4-H ≥ 5 = the number of household members.

All aspects are illustrated in Figure 11.

Figure 11A indicates the statistical differences between FW-0 and FW-1, FW-2 and FW-3 (*p* < 0.0001), FW-1 and FW-3 (*p* < 0.0001), and FW-2 and FW-3 (*p* = 0.000).

Figure 11B shows that the urban zone is substantially correlated with high FW interest (*r* = 0.996, *p* < 0.05), FW perception as money waste (*r* = 0.969, *p* < 0.05), FP daily or every several days (*r* = 0.997, *r* = 0.981, *p* < 0.05), HC weekly, 2–3 times/week, or 2–3 times/month (*r* = 0.987–0.997, *p* < 0.05). The respondents from urban zones are moderately correlated with FW occasionally (*r* = 0.648, *p* > 0.05). Those from rural zones are significantly correlated with very high FW interest (*r* = 0.999, *p* < 0.05) and FP weekly (*r* = 0.976, *p* < 0.05); they also are strongly correlated with HC daily (*r* = 0.923, *p* > 0.05), FP daily (*r* = 0.841, *p* > 0.05), FW money waste, guilty feelings, and FW very rarely (*r* = 0.810–0.830, *p* > 0.05).

Figure 11C displays the correlation between study level and FW interest, FP and HC frequency, and their influence on FW level. All study levels correlate significantly with FW high and very high interest, FP daily, and HC 2–3 times a week (*r* > 0.9, *p* < 0.05). FW very rarely shows a moderate correlation with high school and post-high school (*r* = 0.797, *r* = 0.775, *p* > 0.05).

Figure 11D displays substantial correlations between the number of household members and OSF buying preferences [H1,2,3, and ≥5] with OSFb (frequently, occasionally, never, and rarely, *r* = 0.939–0.996, *p* < 0.05); H4 substantially correlates with OSFb always (*r* = 0.987, *p* < 0.05). Regarding the main reasons for food waste, H2 and H4 significantly correlate with “Food forgotten”, “HMF amount too much”, “Grocery shopping is difficult”, and “HMF leftovers use unknown” (*r* = 0.961–0.989, *p* < 0.05). At the same time, H3 and H ≥ 5 are substantially linked with “No space for HMF preservation” and, respectively, “No time to eat” (*r* = 0.998, *r* = 0.970, *p* < 0.05). H1 and H3 moderately correlate with FW very rarely (*r* = 0.641, *r* = 0.707, *p* > 0.05), while H2 and H4 with FW occasionally (*r* = 0.695, *r* = 0.741, *p* > 0.05). The presence of minor children (<18 years) in the household significantly correlates with OSFb preferences (frequently, occasionally, never, and rarely, *r* = 0.984–0.999, *p* < 0.05), “Food forgotten”, “HMF amount too much”, “Grocery shopping is difficult”, and “HMF leftovers use unknown” (*r* = 0.961–0.984, *p* < 0.05). At the same time, it moderately correlates with FW occasionally (*r* = 0.650, *p* > 0.05).

Figure 11E illustrates the correlations between various age groups and FP behavior, food donation, HFL management, and the main reasons for FW. The respondents of 18–54 years highly correlate with food donation (*r* = 0.919–0.985, *p >* 0.05), while those in the range 55–64, ≥65 show a moderate one (*r* = 0.604, *r* = 0.716, *p* > 0.05). This last age group (≥65) strongly correlates with OSF always, food list respected, HFM freeze and reused, No HFL, FW very rarely (*r* = 0.829–0.997, *p* > 0.05), and moderately correlates with “No food waste” and “No space for HMF preservation” (*r* = 0.657–0.819, *p* > 0.05). The participants aged 18–54 reveal a considerable correlation with “No list”, “Food list relative”, “No space for HMF preservation”, and “HFL saved for the next day” (*r* = 0.879–0.997, *p* > 0.05). The group aged 18–44 moderately correlates with FW occasionally, and 45–54 with FW very rarely (*r* = 0.552–0.714, *p* > 0.05). The heat maps from Figure 11F highlight the main differences between all 4 FW-scored groups.

## 4. Discussion

The adult participants (aged 18 to over 65) in the present study were divided into five age groups according to Employment Rate data indicators [24], aiming to appreciate their financial status. Working-aged people are 18–64 years old: 18–24 is considered an early working age group, 25–54 is prime working, and 55–64 is older working. In 2023, in Romania, the employment rate for early workers (18–24), was 18.7%, while for the older ones (55–64), it was 51.0%. The highest employment rate of the working-age population was registered for graduates of a higher level of education (89.8%), 65.6% of persons with a medium level of education, and 36.9% of low-level ones. The prime workers were divided into three different age groups (25–34, 35–44, and 45–54). Potential differences (involvement in postgraduate studies, family life leading to children < 18 years, more household members, different daily working programs, and incomes) are responsible for FB and FC behaviors and FW level. The age group over 65 includes pensioners—they generally have modest incomes and risks of potential health problems due to aging processes. In the present study, the respondents were personal contacts, colleagues, and relatives of research team members, predominating early and prime workers (93.66%) with high study levels (84.33%) from urban areas (88%).

The factors mentioned above could explain our results regarding FW-level. It is significant (60.30%) in the 18–44 age group and considerably decreases with the age and employment status of participants—from 44.24% in the 45–64 active workers to 14.29% in >65-year-old pensioners. Our results also show that males recorded higher FW levels than females (61.81% vs. 55.91%); the same for urban residency (59.39%) vs. rural (38.23%). Moreover, the FW level increases with study level (48.61% in high school studies vs. 58.31% in bachelor’s degree). All data are similar to those reported in previous studies [25] and fit with the representative individual profile for food waste in Romania: a male consumer under 35 years old, with a high study level of education, living alone in an urban zone [26].

FP depends on a preferred diet. Adopting a healthier, more sustainable diet simplifies the food purchasing process. Planning meals involves a rigorous shopping list and avoiding the impulse to buy. Supporting local food producers through buying local food is a great help for farmers and small businesses in their communities. Understanding food labeling helps to avoid the ones with unhealthy ingredients. The substantial difference between “best before” and “use-by” dates refers to food safety—the “use-by” date inscription means that eating it after that date is not safe for the human body.

In our study, most of the respondents know about food waste (99.33%), understand the concept of “preferably consumed before “(88.66%), and the difference between “to preferably be consumed before” and “to be consumed up to” (80.66%). Almost 66% of them are involved in FP; moderate differences were recorded between the groups with a low frequency of waste food (FW-0 and FW-1) and those with a higher frequency (FW-2 and FW-3): 75% and 66.12% vs. 51.595 and 68.89%. Of the cohort, 16% buy food daily, 42.67% every several days, 36.33% weekly, and 2% every 2 weeks. Most of the FW-0 group buy food weekly (62.50%), followed by FP every 2 weeks (25%) and every several days (12.5%). All FW-1—FW-3 groups buy food daily (19.83% of FW-1, 20.63% of FW-2, and 15.56% of FW-3), every several days (in increasing order, 35.54% of FW-1, 46.83% of FW-2, and 55.56% of FW-3), weekly (in decreasing order, 43.80% of FW-1, 30.16% of FW-2, and 28.89% of FW-3), and every 2 weeks (0.83% of FW-1 and 2.38% of FW-2).

In the present study, of the total cohort, 57.33% have a shopping list but always buy other supplementary groceries, 17.33% have a rigorous shopping list and respect it, and 21.55% do not have a necessary food list. Only 4% declared, “I always buy food on sale, even if I don’t use it right away”. A total of 75% of the FW-0 group have a rigorous shopping list. This percentage drastically decreases in order: FW-1 (26.45%), FW-2 (8.73%), and FW-3 (6.67%). The necessary food list always supplemented with other groceries is used by 45.45% of FW-1, and in similar percentages, by FW-2 and FW-3 (68.25% and 66.67%, respectively). No list stated all FW-1, 2, and 3 in similar percentages (21.49%, 22.22%, and 20.00%). “I always buy food on sale, even if I don’t use it right away”, stated only 0.79% of the FW-2 group and 6.61% and 6.67% of FW-1 and FW-3. Only 2.67% and 13.33% of the total cohort always and frequently buy food on sale. Surprisingly, the highest percentages were recorded in the FW-0 group (12.5% and 25%, respectively). On the other hand, FW-0 has the highest percentage of respondents that “never buy food-on-sale” (37.5% vs. 20.33% of the total cohort). Minimal differences were registered between the total cohort and all other FW groups regarding the frequency of food-on-sale buying.

The European Food Information Council (EUFIC) proposed a series of tools to prevent or diminish FW, consisting of planning meals; knowing food preservation; understanding ‘use by’ vs. ‘best before’ dates; using the available foods; avoiding serving too much; sharing extra food with others; and repurposing waste where possible [27]. Respecting food involves knowing the process that goes into making it. Food production knowledge is a way back to the farmers and their hard work. Food waste means wasting the labor, effort, investment, and resources (water, seeds, feed, etc.) consumed for producing it, as well as the resources for transporting and processing it [28].

Home cooking wastes less food and uses less energy; the environmental impact is minimal when homemade food is mainly based on vegetables. National Resources Defense Council’s report “Wasted” [29] reveals that restaurants produce around 2–4 times the waste of food stores, retail supercenters, and wholesale distributors combined; only 2 percent of the food discarded is donated. People who cook more meals at home, avoid eating out, and have healthier diets with more plant-based and other sustainable food, are less overweight and spend less money on food overall [30]. When farmers and local producers are the principal food sources, home cooking could become sustainable—preparing a meal and eating a dish ensure food waste diminution, suitable protection of the natural environment, and affordable prices [31]. Thus, rural residents’ considerably lower FW levels than urban residents can be explained.

FW interest and education are essential in reducing FW [32]. Various courses are organized for interested people to investigate the causes of food loss and waste and evaluate their effects on the planet (food security, sustainability, and climate change) [33]. Exploring the causes of food waste and suggesting solutions for FW diminution could help to improve the Romanian food system’s sustainability [34].

In the present study, 66.66% of the cohort was interested in FW associated with early measures to reduce FW or desired to know more about FW. In comparison, low and missing interest are more negligible (27.67%, 4.33%, and 1.33%). In the FW-0 and FW-1 groups, the components highlight a great interest in the FW process (62.5% and 25% vs. 48.76% and 31.40%, respectively). FW interest significantly decreased in FW-2 and FW-3 (23.02% and 35.71% vs. 22.22% and 26.67%, respectively). On the other hand, consumers expressed rather negative attitudes towards FW; they felt bad about wasting food and were concerned when they threw food away. As expected, negative emotions are associated with considerable intentions to diminish FW. Negative feelings lead to higher levels of food waste [35]. Consumers are more concerned about the financial consequences of food waste and try to diminish it [35]. Both aspects are evident in our study. FW guilty feelings increase directly proportional to FW frequency, from FW-0 to FW-3 (37.50% to 73.33%).

The perception of FW as a waste of money appears to be constructive, reducing the FW level. The financial level substantially varies with age; age influences food purchase, cooking behavior, and understanding and responsibility toward FW.

Our study shows that the respondents from the urban zone have a great interest in FW, purchase foods daily or every several days, and opt for home cooking weekly, 2–3 times a week, and 2–3 times a month. Those from rural zones have a very high FW interest and prefer FP weekly. The respondents aged >65 always prefer buying food on sale and rigorously respect the necessary food list, probably due to their small incomes. Families with children <18 years old show various preferences for food on sale purchases. They motivate FW by stating that “HMF amount is too much”, “Grocery shopping is difficult”, “Food was forgotten”, and “HMF leftovers use unknown”. The 35–44 age category records the highest FW frequency, followed by 18–24. Similar data were also reported by Dumitru et al. [25].

The results of the present study reveal a substantial FW level (FW-2 + FW-3 = 57%), while 4.5 million Romanian people live in poverty. The main reason for FW was “I forget the food, and it expires before eating” (59.66%), followed by the other two with similar relative frequencies (around 19%): “The amount of Homemade foods is too high for the needs of the household” and “The food has spoiled before the expiry date”. Our results are similar to those of Dumitru et al. [25].

Moreover, the participants evidenced three essential aspects that make diminishing FW difficult: “Grocery shopping is difficult” (46.33%), “I do not have enough space for food preservation” (22.66%), and “I don’t know how to use leftovers” (20%). However, only 37.66% of respondents know about the Food Bank concept, and only 19.66% donate food to the Food Bank; they belong to the active workers group.

Considering all the aspects mentioned earlier, several civil society initiatives have been conducted in different regions of Romania to diminish household food waste [35]. For example, community refrigerators for donated non-perishable food were placed in various zones of Bucharest; they are checked every hour by a General Directorate of Social Assistance guard [26]. For around 15 years, the Vasiliada Association has been running the “Meal of Joy” project in two Romanian counties by partnering with the most known food companies [36]. This association collects commercially non-available food that is 100% safe for consumption and distributes it to needy people [26]. In other cities, social store associations collect food products from households and various companies with their own cars equipped with refrigeration systems and sell them to poor beneficiaries at very low prices [26]. The Romanian Food Banks Federation has nine regional food banks and feeds the people in need with safe food products from over-stocks, mislabeling, and marketing campaigns. Currently, Romanian Food Banks collaborate with 121 donor companies, and since 2016, almost 18.00 tons of food have been distributed to around 225.000 persons [37].

On the other hand, the awareness campaigns combat FW behavior in Romanian customers [38]. The Ministry of Agriculture and Rural Development (MADR) is involved in various projects to educate consumers regarding food loss and waste (awareness-raising campaigns, school activities, seminars and training, and events). Currently, the MADR collaborates with the Ministry of Education for an informal campaign about food waste’s economic, social and environmental impacts [39].

The present study has several limitations. The most significant is that the study database consists of self-reported information on household food waste collected as survey responses. Thus, the sample is not representative of the Romanian population because the selection was probabilistic as subjects wanted to fill in an online form. A few inadvertences were remarked in the no FW group, revealing discrepancies between self-reported data in FW frequency appreciation and detailed food waste investigation. The PCA correlation and heat maps support the mentioned observations. These aspects confirm the relativeness of FW-level analysis based on online questionnaires.

Another limitation is the cohort’s medium size and relative uniformity—due to the limited number and diversity of the individuals who were announced and consequently accessed and completed the online questionnaire. Additionally, some respondent categories are minimally represented: males, people with rural residence, and old age.

## 5. Conclusions

The present study, conducted as an online questionnaire with 300 Romanian participants, offers a complex analysis of socio-demographic factors, behaviors, motivations, and attitudes implied in household food waste management. The results reveal a substantial FW level (57%), showing that homemade food, bread and bakery products, and fruit and vegetables are most wasted. The FW level decreases with people’s income, showing significantly lower values for the old-aged participants. Rural residents also recorded considerably diminished FW levels compared to urban ones. The high involvement of participants in food purchases and homemade cooking is associated with lower food waste. FW decreases when homemade foods are prepared daily. Moreover, the significant interest of participants in FW management can diminish the FW level. However, the FW awareness with negative guilty feelings increases FW, while the economic perception of FW as a money waste is constructive and decreases FW level.

All these observations have potential applications in further food waste projects. Future studies could deeply analyze the impact of spiritual, emotional, and behavioral factors in more different and numerous communities. Moreover, understanding why people waste food and identifying the essential difficulties in the fight against food waste is only the beginning. FW is an emergency; future studies may find sustainable solutions to reduce its long-term harmful effects.

## Figures and Tables

**Table 1:** Socio-demographic characteristics of all 300 respondents and FW-scores.

Parameter	Category	T		F		M		*p*-Value		
Frequency/Relative Frequency		N	%	N	%	N	%	T/F	F/M	T/M
Age (years)	Total	300.00	100.00	245.00	81.67	55.00	18.33	0.68	0.04 *	0.04 *
	18–24	98.00	32.67	82.00	33.47	16.00	29.09			
	25–34	33.00	11.00	28.00	11.43	5.00	9.09			
	35–44	103.00	34.33	80.00	32.65	23.00	41.82			
	45–54	47.00	15.67	40.00	16.33	7.00	12.73			
	55–64	12.00	4.00	9.00	3.67	3.00	5.45			
	≥65	7.00	2.33	6.00	2.45	1.00	1.82			
Study level	Bachelor’s degree	172.00	57.33	140.00	57.14	32.00	58.18	0.77	0.16	0.14
	High school	36.00	12.00	28.00	11.43	8.00	14.55			
	Post-high school	10.00	3.33	9.00	3.67	1.00	1.82			
	Postgraduate	82.00	27.33	68.00	27.76	14.00	25.45			
Residence	Rural	34.00	11.33	27.00	11.02	7.00	12.73	0.87	0.43	0.40
	Urban	266.00	88.67	218.00	88.98	48.00	87.27			
Family members number	1	46.00	15.33	33.00	13.47	13.00	23.64	0.53	0.01 *	0.01 *
	2	93.00	31.00	79.00	32.24	14.00	25.45			
	3	85.00	28.33	68.00	27.76	17.00	30.91			
	4	54.00	18.00	46.00	18.78	8.00	14.55			
	≥5	22.00	7.33	19.00	7.76	3.00	5.45			
Children < 18 years	No	134.00	44.67	109.00	44.49	25.00	45.45	0.31	0.02 *	0.02 *
	Yes	166.00	55.33	136.00	55.51	30.00	54.55			
FW Score	FW-0	8.00	2.67	7.00	2.86	1.00	1.82	0.72	0.09	0.08
	FW-1	121.00	40.33	101.00	41.22	20.00	36.36			
	FW-2	126.00	42.00	99.00	40.41	27.00	49.09			
	FW-3	45.00	15.00	38.00	15.51	7.00	12.73			

* *p* < 0.05 = statistically significant differences; N = number (frequency); % = relative frequency; FW = food waste. FW-0 = never; FW-1 = very rarely; FW-2 = occasionally; FW-3 = frequently; T = total; M = male; F = female.

## Data Availability

Data are available in the article and Supplementary Materials.

## Supplementary Materials

The following supporting information can be downloaded at https://www.mdpi.com/article/10.3390/nu16162738/s1. Table S1: Validation of the questionnaire using Reliability Analysis from XLSTAT; Table S2: Food purchasing and homemade food cooking variables; Table S3: Food waste linked variables; Table S4: Knowledge, feelings, motivation, and food waste frequency.
