# Supplementary material for: Socio-Demographic Factors, Behaviors, Motivations, and Attitudes in Food Waste Management of Romanian Households
Source: Nutrients. 2024 Aug 16;16(16):2738. doi: 10.3390/nu16162738 (PMC11356938; doi:10.3390/nu16162738)
Supplement: Supplementary file 1 [file nutrients-16-02738-s001.zip › nutrients-3129615-supplementary.pdf]

nutrients

MDPI

Supplementary Material

Table S1. Validation of the questionnaire using Reliability Analysis from XLSTAT:

Cronbach's alpha statistics :		
Cronbach's alpha	Standardized Cronbach's Alpha	
0,990	0,998	
1. Cronbach's alpha statistics Split-Half 1:		
Cronbach's alpha	Standardized Cronbach's Alpha	
0,983	0,997	
2. Cronbach's alpha statistics Split-Half 2:		
Cronbach's alpha	Standardized Cronbach's Alpha	
0,977	0,996	
Split-Half statistics:		
Split-Half correlation	Spearman-Brown coefficient	Guttman L4
0,999	0,999	0,998

Table S2. Food purchasing and homemade food cooking variables.

Parameter	FW Score	FW-0		FW-1		FW-2		FW-3	
	Frequency/relative frequency	N	%	N	%	N	%	N	%
Food purchasing (FP) frequency	FP Daily	0.00	0.00	24.00	19.83	26.00	20.63	7.00	15.56
	FP Every two weeks	2.00	25.00	1.00	0.83	3.00	2.38	0.00	0.00
	FP Several days	1.00	12.50	43.00	35.54	59.00	46.83	25.00	55.56
	FP Weekly	5.00	62.50	53.00	43.80	38.00	30.16	13.00	28.89
FP involvement	FP Shared	2.00	25.00	38.00	31.40	59.00	46.83	13.00	28.89
	FP No implied	0.00	0.00	3.00	2.48	2.00	1.59	1.00	2.22
	FP Very implied	6.00	75.00	80.00	66.12	65.00	51.59	31.00	68.89
Food buying behavior	Food list relative	1.00	12.50	55.00	45.45	86.00	68.25	30.00	66.66
	Food list respected	6.00	75.00	32.00	26.45	11.00	8.73	3.00	6.66
	No list	1.00	12.50	26.00	21.48	28.00	22.22	9.00	20.00
	OSF always	0.00	0.00	8.00	6.61	1.00	0.79	3.00	6.66
BBD knowledge status	BBD – Not known	1.00	12.50	8.00	6.61	10.00	7.94	5.00	11.11
	BBD - No	0.00	0.00	3.00	2.48	7.00	5.56	0.00	0.00
	BBD - Yes	7.00	87.50	110.00	90.91	109.00	86.51	40.00	88.89
Food-on-sale buying (OSFb)	OSFb Always	1.00	12.50	2.00	1.65	3.00	2.38	2.00	4.44
	OSFb Frequently	2.00	25.00	17.00	14.05	16.00	12.70	5.00	11.11

frequency	OSFb Never	3.00	37.50	25.00	20.66	25.00	19.84	8.00	17.78
	OSFb Occasionally	2.00	25.00	45.00	37.19	45.00	35.71	19.00	42.22
	OSFb Very rarely	0.00	0.00	32.00	26.45	37.00	29.37	11.00	24.44
Homemade food cooking (HC) involvement	HC No implied	0.00	0.00	10.00	8.26	19.00	15.08	6.00	13.33
	HC Share	2.00	25.00	37.00	30.58	49.00	38.89	16.00	35.56
	HC Very implied	6.00	75.00	74.00	61.16	58.00	46.03	23.00	51.11
HC frequency	HC Daily	3.00	37.50	36.00	29.75	31.00	24.60	6.00	13.33
	HC Two/three times a month	0.00	0.00	6.00	4.96	8.00	6.35	1.00	2.22
	HC Two/three times a week	4.00	50.00	59.00	48.76	63.00	50.00	31.00	68.89
	HC Very rarely	0.00	0.00	3.00	2.48	3.00	2.38	3.00	6.67
	HC Weekly	1.00	12.50	17.00	14.05	21.00	16.67	4.00	8.89

FP = food purchase, FC = food cooking, BBD = best before date, OSF = food-on-sale (with a low BBD), OSFb = food on sale buying; HC = home cooking, FW = food waste.

Table S3. Food waste linked variables.

Parameter	FW Score Frequency/relative frequency	FW-0		FW-1		FW-2		FW-3	
		N	%	N	%	N	%	N	%
Leftovers of Homemade Foods (HFL) management	HFL freezed	0.00	0.00	5.00	4.13	0.00	0.00	0.00	0.00
	HFL reused	0.00	0.00	3.00	2.48	1.00	0.79	0.00	0.00
	HFL saved for next day	6.00	75.00	96.00	79.34	111.00	79.34	34.00	75.56
	HFL throwed away	0.00	0.00	0.00	0.00	7.00	5.56	3.00	6.67
	HFL used for animal feed	0.00	0.00	4.00	3.31	4.00	3.17	8.00	17.78
	No HFL	2.00	25.00	13.00	10.74	3.00	2.38	0.00	0.00
FW term knowledge status	FW Kn No	0.00	0.00	0.00	0.00	1.00	0.79	1.00	2.22
	FW Kn Yes	8.00	100.00	121.00	100.00	125.00	99.21	44.00	97.78
Food waste information source	FWi - F/f	3.00	37.50	11.00	9.09	12.00	9.52	3.00	6.67
	FWi - Not known	0.00	0.00	0.00	0.00	1.00	0.79	1.00	2.22
	FWi - Mm	5.00	62.50	89.00	73.55	95.00	75.40	32.00	71.11
	FWi - Sm	0.00	0.00	21.00	17.36	18.00	14.29	9.00	20.00
The main reasons for food waste	Food forgotten	2.00	25.00	57.00	47.11	87.00	69.05	33.00	73.33
	HMF amounts to too much	1.00	12.50	19.00	15.70	26.00	20.63	10.00	22.22
	Food spoiled before bbd	1.00	12.50	43.00	35.54	13.00	10.32	2.00	4.44
	No food waste	4.00	50.00	2.00	1.65	0.00	0.00	0.00	0.00
The reasons	Grocery shopping difficult	2.00	25.00	48.00	39.67	67.00	53.17	22.00	48.89

that make difficult the food waste diminution	HMF leftovers use unknown	0.00	0.00	21.00	17.36	29.00	23.02	10.00	22.22
	HMF refrigerating unknown	0.00	0.00	6.00	4.96	2.00	1.59	0.00	0.00
	No food waste	4.00	50.00	10.00	8.26	0.00	0.00	1.00	2.22
	No space for HMF preservation	1.00	12.50	32.00	26.45	23.00	18.25	12.00	26.67
	No time for eating	1.00	12.50	4.00	3.31	5.00	3.97	0.00	0.00
FW interest of people	FW interest high	2.00	25.00	38.00	31.40	45.00	35.71	12.00	26.67
	FW interest moderate	1.00	12.50	21.00	17.36	40.00	31.75	21.00	46.67
	FW interest very high	5.00	62.50	59.00	48.76	29.00	23.02	10.00	22.22
	No FW interest	0.00	0.00	1.00	0.83	3.00	2.38	0.00	0.00
	FW interest low	0.00	0.00	2.00	1.65	9.00	7.14	2.00	4.44
Status of personal involvement in donating food	Food donation No	6.00	75.00	94.00	77.69	104.00	82.54	37.00	82.22
	Food donation Yes	2.00	25.00	27.00	22.31	22.00	17.46	8.00	17.78
Food waste Awareness status	FW not significant	1.00	12.50	4.00	3.31	5.00	3.97	1.00	2.22
	FW guilty feelings	3.00	37.50	71.00	58.68	78.00	61.90	33.00	73.33
	FW = money waste	4.00	50.00	46.00	38.02	42.00	33.33	11.00	24.44
	FW can not be reduced	0.00	0.00	0.00	0.00	1.00	0.79	0.00	0.00

N = Frequency (number); % = relative frequency; FW = food waste; kn = knowledge; FWi = food waste information; F/f = family and friends, Mm = mass media; Sm = social media; HMF = homemade food; HFL = homemade food leftovers.

Table S4. Knowledge, feelings, motivation, and food waste frequency.

Parameter	Food waste in the last seven days	I don't know		No		Yes	
		N	%	N	%	N	%
	Total	15.00	5.00	106.00	35.33	179.00	59.67
Food waste frequency	Frequently (FW-3)	1.00	6.67	18.00	16.98	26.00	14.53
	Never (FW-0)	0.00	0.00	2.00	1.89	6.00	3.35
	Occasionally (FW-2)	8.00	53.33	56.00	52.83	62.00	34.64
	Very rarely (FW-1)	6.00	40.00	30.00	28.30	85.00	47.49
The main reasons for food waste	I forget the food, and then it expires before I eat.	7.00	46.67	51.00	48.11	121.00	67.60
	The amount of homemade foods is too high for the household's needs.	2.00	13.33	18.00	16.98	36.00	20.11
	The food has spoiled before the expiry date	6.00	40.00	31.00	29.25	22.00	12.29

The reasons for making difficult food waste diminution	Grocery shopping is difficult	8.00	53.33	36.00	33.96	95.00	53.07
	I don't know how to use leftovers	4.00	26.67	19.00	17.92	37.00	20.67
	I don't know the rules for keeping food in the refrigerator.	0.00	0.00	7.00	6.60	1.00	0.56
	I don't throw away food	0.00	0.00	12.00	11.32	3.00	1.68
	I have not enough space for food preservation	3.00	20.00	27.00	25.47	38.00	21.23
	No time for eating	0.00	0.00	5.00	4.72	5.00	2.79
Frequently waisted food types	Bread and bakery products	4.00	26.67	20.00	18.87	57.00	31.84
	Eggs	0.00	0.00	1.00	0.94	0.00	0.00
	Fruits and vegetables	3.00	20.00	19.00	17.92	21.00	11.73
	Homemade foods	3.00	20.00	20.00	18.87	66.00	36.87
	Milk-derivatives	2.00	13.33	11.00	10.38	18.00	10.06
	No wasted foods	3.00	20.00	29.00	27.36	1.00	0.56
	Raw meat	0.00	0.00	1.00	0.94	4.00	2.23
	Sausages	0.00	0.00	5.00	4.72	12.00	6.70
FW interest of people	I care, and I want to know more about the impact of food waste.	4.00	26.67	36.00	33.96	57.00	31.84
	I care, but it is not very important to me.	6.00	40.00	18.00	16.98	59.00	32.96
	I care so much, and I try to take measures to reduce food waste.	5.00	33.33	46.00	43.40	52.00	29.05
	I don't care	0.00	0.00	2.00	1.89	2.00	1.12
	It is not very important to me.	0.00	0.00	4.00	3.77	9.00	5.03
"Food bank" concept knowledge status	No	8.00	53.33	111.00	62.01	241.00	80.33
	Yes	7.00	46.67	68.00	37.99	59.00	19.67
Status of personal involvement in donating food to food banks	No	13.00	86.67	85.00	80.19	143.00	79.89
	Yes	2.00	13.33	21.00	19.81	36.00	20.11
Understanding of the difference between "to preferably be consumed before" and "to be consumed up to"	No	2.00	13.33	22.00	20.75	34.00	18.99
	Yes	13.00	86.67	84.00	79.25	145.00	81.01
Understanding the concept of "preferably consumed before"	I don't know	2.00	13.33	3.00	2.83	19.00	10.61
	No	0.00	0.00	6.00	5.66	4.00	2.23
	Yes	13.00	86.67	97.00	91.51	156.00	87.15
Food waste awareness status	Food waste is not a significant problem for me	1.00	6.67	4.00	3.77	6.00	3.35
	Food waste makes me feel guilty	6.00	40.00	61.00	57.55	118.00	65.92
	I believe that wasting food is a waste of money	8.00	53.33	41.00	38.68	54.00	30.17

	I can't reduce the amount of wasted food because of the other household members	0.00	0.00	0.00	0.00	1.00	0.56
Age	18-24	6.00	40.00	32.00	30.19	60.00	33.52
	25-34	2.00	13.33	9.00	8.49	22.00	12.29
	35-44	4.00	26.67	34.00	32.08	65.00	36.31
	45-54	2.00	13.33	20.00	18.87	25.00	13.97
	55-64	1.00	6.67	5.00	4.72	6.00	3.35
	≥65	0.00	0.00	6.00	5.66	1.00	0.56
Gender	F	14.00	93.33	84.00	79.25	147.00	82.12
	M	1.00	6.67	22.00	20.75	32.00	17.88
Studies	bachelor degree	6.00	40.00	59.00	55.66	107.00	59.78
	high school	3.00	20.00	16.00	15.09	17.00	9.50
	post-high school	0.00	0.00	2.00	1.89	8.00	4.47
	postgraduate	6.00	40.00	29.00	27.36	47.00	26.26
Residency	Rural	3.00	20.00	9.00	8.49	22.00	12.29
	Urban	12.00	80.00	97.00	91.51	157.00	87.71

FW = food waste; kn = knowledge; FWi = food waste information; F/f = family and friends; Mm = mass media; Sm = social media; HMF = homemade food; HFL = homemade food leftovers.
